# Understanding Horizontal Gene Transfer network in human gut microbiota

**DOI:** 10.1186/s13099-020-00370-9

**Published:** 2020-07-09

**Authors:** Chen Li, Jiaxing Chen, Shuai Cheng Li

**Affiliations:** Department of Computer Science, City University of Hong Kong, Hong Kong, Hong Kong SAR, China

**Keywords:** HGT network, Scale free, Von Newman entropy, Network evolving, Community analysis

## Abstract

**Background:**

Horizontal Gene Transfer (HGT) is the process of transferring genetic materials between species. Through sharing genetic materials, microorganisms in the human microbiota form a network. The network can provide insights into understanding the microbiota. Here, we constructed the HGT networks from the gut microbiota sequencing data and performed network analysis to characterize the HGT networks of gut microbiota.

**Results:**

We constructed the HGT network and perform the network analysis to two typical gut microbiota datasets, a 283-sample dataset of Mother-to-Child and a 148-sample dataset of longitudinal inflammatory bowel disease (IBD) metagenome. The results indicated that (1) the HGT networks are scale-free. (2) The networks expand their complexities, sizes, and edge numbers, accompanying the early stage of lives; and microbiota established in children shared high similarity as their mother (p-value = 0.0138), supporting the transmission of microbiota from mother to child. (3) Groups harbor group-specific network edges, and network communities, which can potentially serve as biomarkers. For instances, IBD patient group harbors highly abundant communities of *Proteobacteria* (p-value = 0.0194) and *Actinobacteria* (p-value = 0.0316); children host highly abundant communities of *Proteobacteria* (p-value = 2.8785$e^{-5}$) and *Actinobacteria* (p-value = 0.0015), and the mothers host highly abundant communities of *Firmicutes* (p-value = 8.0091$e^{-7}$). IBD patient networks contain more HGT edges in pathogenic genus, including *Mycobacterium*, *Sutterella*, and *Pseudomonas*. Children’s networks contain more edges from *Bifidobacterium* and *Escherichia*.

**Conclusion:**

Hence, we proposed the HGT network constructions from the gut microbiota sequencing data. The HGT networks capture the host state and the response of microbiota to the environmental and host changes, and they are essential to understand the human microbiota.

## Introduction

Human gut microbiota is a complex ecosystem consisting of a total of 10$^{14}$ bacteria [1]. Species richness makes gut microbiota harbor diverse metabolic functions and robust to disturbances, such as the invasion of pathogenic bacteria while maintaining host health. However, factors [2], such as age [3], environment [4], diet [5], can lead to significant shifts in the composition of the individual microbiome over longitudinal periods. Microbes may share genetic materials with others to get beneficial traits through Horizontal Gene Transfer (HGT) to gain better adaptions. HGTs allow microbes to acquire genes from distant species that are not in a parent–offspring relationship [6]. It increases the genetic diversity of recipients and plays a vital role in the evolution of microbes. The complex interaction among species indicates that gut microbiota is a complex system.

Researchers have developed tools to detect HGTs. GIST [7] identified HGT events by searching for regions with different genomic signatures from the genome average, and it can detect HGT among distantly related genomes. While for some ancient HGT events, genomic signatures are similar to background subjected to the mutation process [8] and are hard to be recognized by GIST. Darkhorse [9] and HGTector [10] identified HGT events in genomes showing taxonomically discordant similarity to genes within a reference database. Compared to GIST, Darkhorse and HGTector are biased toward more ancient HGT events and not make use of phylogenetic trees to test for phylogenetic incongruence. MetaCHIP [11] identified HGT events based on best-match and phylogenetic incongruency approaches [12]. The main challenge of MetaCHIP is to detect recent HGT events [13]. Essentially, HGT is the insertion of foreign genes into recipient genomes. It is similar to interchromosomal translocation in an organism with multiple chromosomes [14]. Therefore, HGT can be treated as a kind of complicated structure variation (SV). No matter ancient or recent HGT events could be identified by mapping NGS reads against reference genomes. LEMON [15] applies split reads re-alignment [16] and DBSCAN [17] to detect HGT events.

Network science is a powerful tool to analyze and understand such complex systems. Various mathematical models are available to analyze and quantify such systems [18–22]. In order to use networks to model the human gut microbiota, one method calculates the correlation coefficients for the abundances of each pair microbes. The correlation matrix forms a network [23]. The positive and negative correlation indicates the two microbes may have cooperation or competition interact, respectively. However, this network model is incapable of capturing the potential mechanism for the interactions. Another method is based on the metabolic exchange between microbes to construct the Global metabolic interaction network of the human gut microbiota [24]. It requires annotations of enzymes and metabolic pathways. The HGT networks represent how human gut, oral, nasopharyngeal skin microbiomes share genetic material to adapt to the environment. They provide new insights into the dynamics of the microbiota. Through analyzing the HGT networks, we may gain new insights into the communities assemble, species interactions, and host-associated selection pressure on the microbiota. Kunin et al. [25] constructed the HGT network based on the reconstructed phylogenetic trees. It utilized protein sequences as evolutionary units, limiting its ability to detect HGT events in regions outside or across gene boundaries [26]. While these HGT events could be detected by LEMON, which takes whole metagenomic sequencing data as input, thus, LEMON could be used to create a complete HGT network.

We apply the HGT networks to study two typical human gut microbiota datasets.

The first is a 283-sample dataset of Mother-to-Child [3]. The development of infant gut microbiota plays an important role in establishing a healthy host–microbiome symbiosis, including the maturation of the immune system [27], nutrient utilization [28], and so on. The dynamic microbiota grows rapidly and is affected by factors such as delivery mode [29] and feeding [30]. As the importance of infant gut microbiota has been realized, how and where an infant acquires these microbes attract increasing attention. The maternal gut bacteria is considered as one important source, but the vertical inheritance remains largely unexplored. Ferretti et al. [31] utilized strain-level metagenomic profiling to track the mother-to-infant bacterial transmission; they find strains shared within the mother and infant pairs. However, their research is unable to capture the significant similarity of the gut microbiome in mother–child pairs by using Bray–Curtis dissimilarity.

The second is a 148-sample dataset of longitudinal Inflammatory bowel disease (IBD) metagenome [32]. The composition of gut microbiota is related to many diseases [33], such as IBD [34] and type 1 diabetes [35]. IBD is one most common groups of chronic inflammatory disorders affecting millions of people. The cause of IBD is associated with human genetic mutation and gut microbiota. The gut microbiota plays an important role in IBD [36]. The increasing of *Bacteroidetes* and decreasing of *Firmicutes* are observed in IBD patients [37]. Since gut microbiota is dynamic, the longitudinal gut microbiota is analyzed to capture the variation of gut microbiota composition over time in IBD patients [38]. The linkage between metagenomic functional potential and functional activity is built in recent research [32]. However, these work fail to model gut microbiota from a systematic perspective, which motivates us to apply HGT network analysis.

Our results consist of three aspects. First, we investigate the general characteristics of the HGT network. HGT network is a complex network, and we used power-law distribution and three heavy tail distributions to fit the network. The result demonstrates that the HGT networks are scale-free, which implies the HGT network holds important properties such as ultra-small world property and robust to random disruption. Second, we studied the dynamic change of HGT networks, especially for the Mother-to-Child data set. The increasing complexity (Von Newman Entropy), network size, and HGT event rate in child HGT networks accompany the growth of child gut microbiota during the first three months after birth. Furthermore, the high structural similarity (p-value = 0.0138) between the child and maternal HGT networks supports the transmission of microbiota from mother to child. Third, we analyze phenotype-specific HGT communities and HGT edges. As for HGT communities, compared with control individuals, the phylum composition of IBD-specific HGT communities have significant increasing of *Proteobacteria* (p-value = 0.0194) and *Actinobacteria* (p-value = 0.0316). Compared with mother, the phylum composition of child-specific HGT communities have significant decreasing of *Firmicutes* (p-value = 8.0091$e^{-7}$) [39] and increasing of *Proteobacteria* (p-value = 2.8785$e^{-5}$) and *Actinobacteria* (p-value = 0.0015) [40, 41]. As for conserved HGT edges across multiple samples, IBD patients have increased HGT edges in pathogenic genus including *Mycobacterium*, *Sutterella*, and *Pseudomonas*, compared to non-IBD individuals. Child-specific HGT edges are mainly from *Bifidobacterium* and *Escherichia*. These differences imply the alteration of gut microbiota caused by the change of selection pressure.

As we can see, both in Mother-to-Child and longitudinal IBD data sets, by analyzing temporal HGT networks, we captured the significant changes of HGT networks. These changes reflect the alteration of gut microbiota under the change of host-associated selection pressure. Therefore, the HGT network is an effective model to describe the relationship between the gut microbiota and the host state. It provides a new perspective to observe the change of gut microbiota in the everchanging environment.

## Results

We applied HGT networks analysis to two human gut longitudinal metagenomic sequencing datasets: Mother-to-Child data set [3] and longitudinal IBD data set [32]. As described in [3], the Mother-to-Child data set contains 283 samples that are collected from 44 Finish families. Thirty-three families have children sampled at five-time points: birth, two weeks, and one, two, and three months and mother sampled at three-time points: gestational week 27, birth, and three months post-delivery. The remaining 11 families have children sampled at birth and mother sampled at gestational week 27 and birth. As described in [32], the longitudinal IBD data set contains 148 samples spanning 26 participants: 15 patients with Crohn’s disease (CD), eight patients with ulcerative colitis (UC) and three non-IBD controls, here CD and UC are two main forms of IBD.

**Fig. 1:**
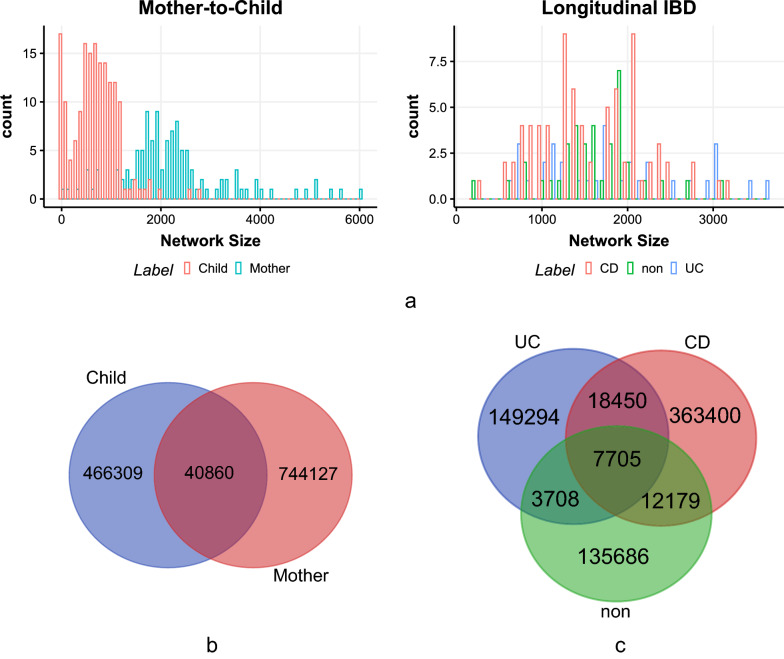
**a** Distribution of HGT network size in Mother-to-Child data set and longitudinal IBD data set; **b** and **c** Venn diagrams of HGT events identified in Mother-to-Child and IBD data sets respectively. **b** The number of shared HGT events is 40,860, while the number of Child-specific and Mother-specific HGT events are 466,309 and 744,127 respectively. **c** The number of HGT events shared by UC, CD, and non-IBD controls is 7,705, while the number of UC-specific, CD-specific, and non-IBD-specific HGT events are 149,294, 363,400, and 135,686 respectively

Following the method in, we constructed one HGT network per sample. Then we obtained 283 HGT networks from the Mother-to-Child data set and 148 HGT networks from the longitudinal IBD data set. Figure 1a illustrates the distribution of the size of HGT networks constructed from the two datasets. Network size is the number of nodes in the HGT network. The averagechild form individual network size of infant HGT networks is 702.57, and the average network size of maternal HGT networks is 2228.12. There is an overlap between the distribution of network size for the HGT networks detected from CD, UC, and non-IBD controls individuals. Their average network sizes are 1604.71, 1795.03, and 1611.66, respectively. Figure 1b, c are Venn diagrams of HGT events identified in Mother-to-Child and IBD data sets, respectively. Common HGT events shared by different groups are much less than group-specific HGT events. In the Mother-to-Child data set, the number of shared HGT events is 40,860, while the number of Child-specific and Mother-specific HGT events are 466,309 and 744,127, respectively. In Longitudinal IBD, the number of HGT events shared by UC, CD, and non-IBD controls is 7705, while the numbers of UC-specific, CD-specific, and non-IBD-specific HGT events are 149,294, 363,400, and 135,686, respectively. So, HGT event overlap could effectively measure the overlap between HGT networks under different conditions.

### HGT networks are scale free

Our study implies that the HGT network is scale-free. It is supported by the result that the degree distribution of the HGT network is better fitted by power-law than the other three heavy tail distribution [42]. We filtered out HGT networks with less than 100 nodes to ensure the remaining HGT networks have enough degree data to fit. Finally, we collected 256 HGT networks from the Mother-to-Child data set and 147 HGT networks from the longitudinal IBD data set. We applied *powerlaw* package [43] to estimate degree distribution of HGT networks.

**Fig. 2:**
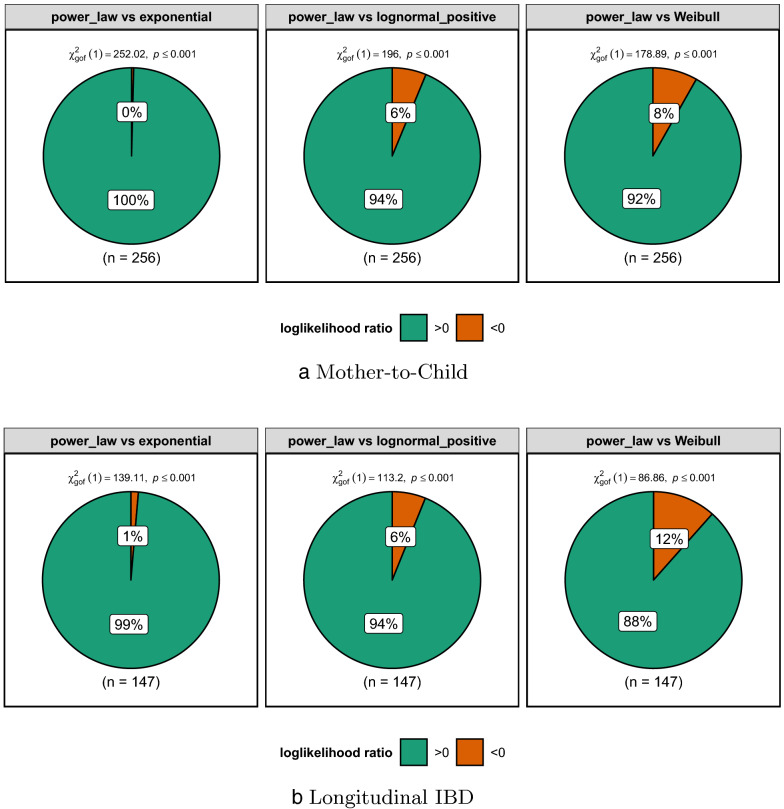
**a** Loglikelihood ratio test for powerlaw vs exponential and powerlaw vs lognormal_positive fitting to HGT networks of Mother-to-Child data set; **b** Loglikelihood ratio test for powerlaw vs exponential and powerlaw vs lognormal_positive fitting to HGT networks of Longitudinal IBD data set. In each test, if the ratio is larger than 0, the power law achieves better fit than the other

The evaluation of the goodness of fit for power-law distribution is described in “Method” section. As illustrated in Fig. 2a, 100%, 94%, and 92% of HGT networks are better fitted by power-law than exponential, lognormal_positive, and Weibull respectively in Mother-to-Child data set. Meanwhile, Fig. 2b demonstrates that in Longitudinal IBD data set 99%, 94%, and 88% of HGT networks are better fitted by power-law than exponential, lognormal_positive, and Weibull. Therefore, the HGT network is scale-free since HGT networks have degree distribution better fitted by power-law than the other three heavy tail distributions. Besides, as we can see in Fig. 5, a vast number of nodes of the HGT network has a small degree. They are connected to a few hub nodes. These hub nodes would have a very large degree. Thus, the degree of a randomly selected node would be tiny or arbitrarily large, which means HGT networks do not have a meaningful internal scale [44]. It explains the scale-free property of the HGT network.

**Fig. 3:**
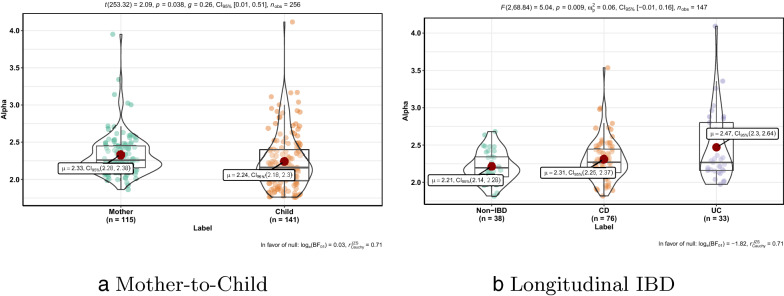
**a** Comparison of exponents *alpha* fitted to maternal and child HGT networks respectively; **b** Comparison of exponents *alpha* fitted to HGT networks of non-IBD, UC, and CD respectively

HGT networks in different groups harbor different distributions of fitted exponents *alpha* which is the parameter of power-law distribution. Figure 3a compares exponents *alpha* fitted to maternal and child HGT networks respectively. The distribution of fitted exponents corresponding to Mother is significantly different to the one corresponding to child (p = 0.038, Student’s t-test) and has larger mean value $\mu _{mother}>\mu _{child}$. Figure 3b compares exponents *alpha* fitted to HGT networks of non-IBD, UC, and CD respectively. Three exponent distributions are significantly different to each other (p = 0.009, Student’s t-test) and have $\mu (UC)>\mu (CD)>\mu (Non$-*IBD*).

As shown in Fig. 3, the 95% Confidence Intervals $CI_{95\%}$ of fitted exponent *alpha* in all groups are in region (2, 3). So HGT networks hold the ultra-small world property, which implies that HGT networks tend to form dense sub-graphs. Such a network structure implies that the HGT network is robust and could maintain a stable status [45].

**Fig. 4:**
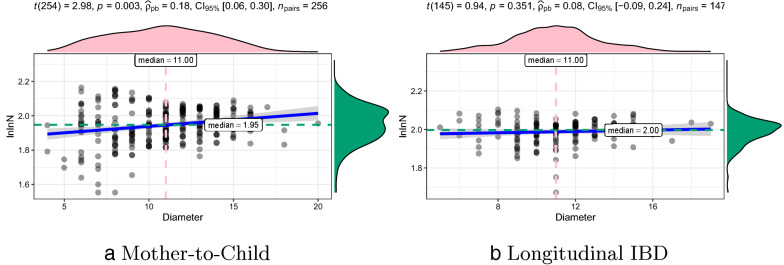
The two scatter plots shows the linear relationship between *d* and *lnlnN* of HGT networks in Mother-to-Child and Longitudinal IBD data sets. This demonstrates the ultra-small world property of HGT networks

### Ultra-small world property of HGT networks

Since the degree exponent $\alpha$ of the HGT network satisfies $2<\alpha <3$, HGT networks have the property of the ultra-small world [46]. The ultra-small world property means that the diameter *d* of HGT networks has a linear relationship with *lnlnN*, where *N* is the number of nodes in the network, *ln* is the natural logarithm. Figure 4 shows the linear relationship between *d* and *lnlnN* of HGT networks in Mother-to-Child and Longitudinal IBD data sets. The ultra-small diameter *d* increases as *lnlnN*, which is significantly slower growth than the *lnN* derived for random networks. Therefore, the average distance in an HGT network is smaller than that in a random network, which imply the ultra-small world property of HGT networks.

**Fig. 5:**
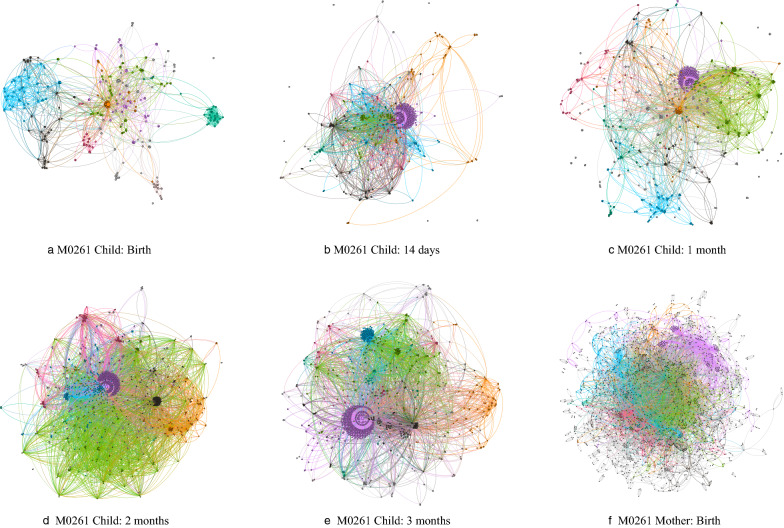
Evolvement of HGT networks in family M0261. **a**–**e** The temporal evolvement of the Child HGT network. **f** The maternal HGT network at birth. Each node represents a reference genome. Each edge denotes the existence of HGT events between two reference genomes

### Analysis of Mother-to-Child HGT networks

#### HGT network evolves

We studied the evolvement of HGT networks across time from two aspects: network complexity and network similarity. Network complexity is measured by Von Newman entropy, network size, and HGT event rate. Network similarity is measured by the Jaccard index and degree correlation. We observed, first, child HGT networks have increasing network complexity in the first three months after birth. Second, maternal and child HGT networks share a family-specific significant similarity. Last, the individual-specific similarity of the HGT network between different time points in IBD patients is significantly larger than that in non-IBD individuals. The evolvement of the infant HGT network describes the growth of gut microbiota. Figure 5 shows the temporal evolvement of child HGT networks across five-time points and the maternal HGT network at birth. As time goes by, the child HGT network becomes bigger and more complex due to the growth of child intestinal microbial strains harboring HGT events. Compared to child HGT networks, the maternal HGT network contains more nodes and edges, so it has a more complex internal structure.

**Fig. 6:**
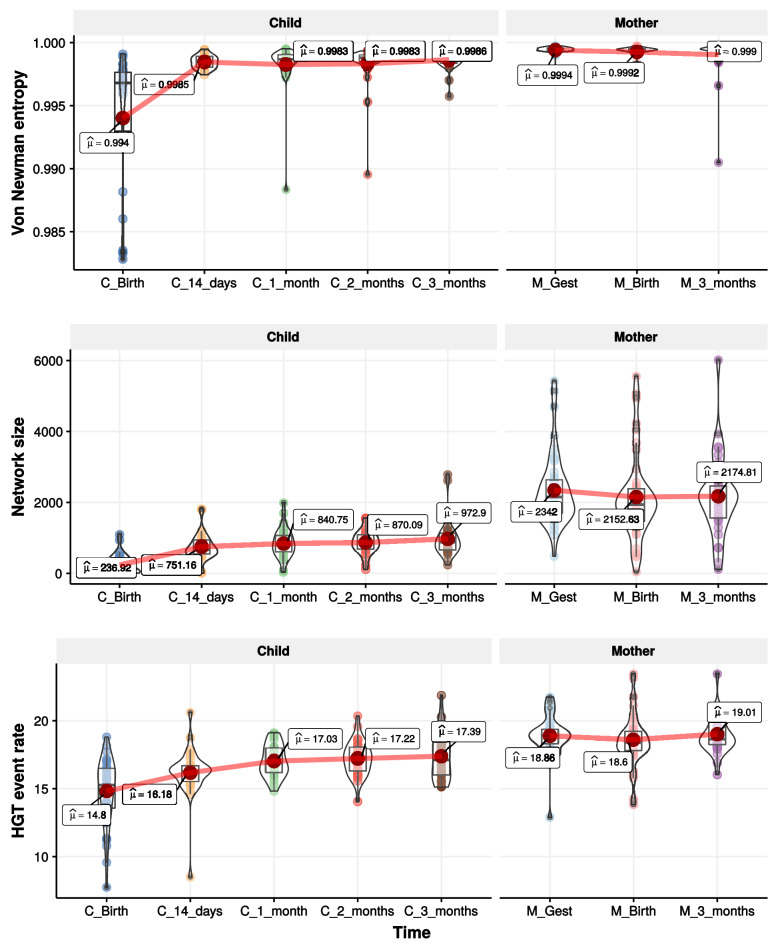
The complexity evolvement of maternal and child HGT networks during the first three months are measured from three aspects: Von Newman Entropy, Network size, and HGT event rate

We analyzed the evolvement of maternal HGT networks at three-time points: gestational week 27 (M_Gest), birth (M_Birth), and three months post-delivery (M_3_months), and child HGT networks during the first three months. The evolvement is measured from three aspects: Von Newman entropy, network size, and HGT event rate. The Von Newman Entropy measures the complexity of the network (see “Method” section) Network size is the number of nodes in the HGT network. HGT event rate is defined in “Method” section . It measures the number of HGT events detected in one sample. We calculate the three metrics for all HGT networks at each time point. As shown in Fig. 6, for child HGT networks, the three metric keep increasing during the first three months, which imply the increasing of the number of HGT events and the growing number of strains involved in HGT events. Compared to the evolvement of child HGT networks, the three metrics of maternal HGT networks do not have increasing trends and maintain a relatively high level at the three-time points. This reflects the stability of mature gut microbiota. Furthermore, the increasing trends of the Von Newman Entropy, Network size, and HGT event rate of child HGT networks indicate the rapid growth of child gut microbiota during the first three months after birth. The average Von Newman Entropy of child HGT networks rise from 0.994 to 0.9983. The average network size rises from 236.92 to 972.9, and the average HGT event rate rises from 14.8 to 17.39. This growth process could be described by the evolvement of the HGT network. It usually takes 2 or 3 years to achieve the established microbiota among children [47]. To study the establishing of microbiota in child, it is worthwhile to analyze the evolvement of the HGT network after three months, which could be in our future work (Fig. 6).

#### Family-specific similarity of maternal and child HGT networks

**Fig. 7:**
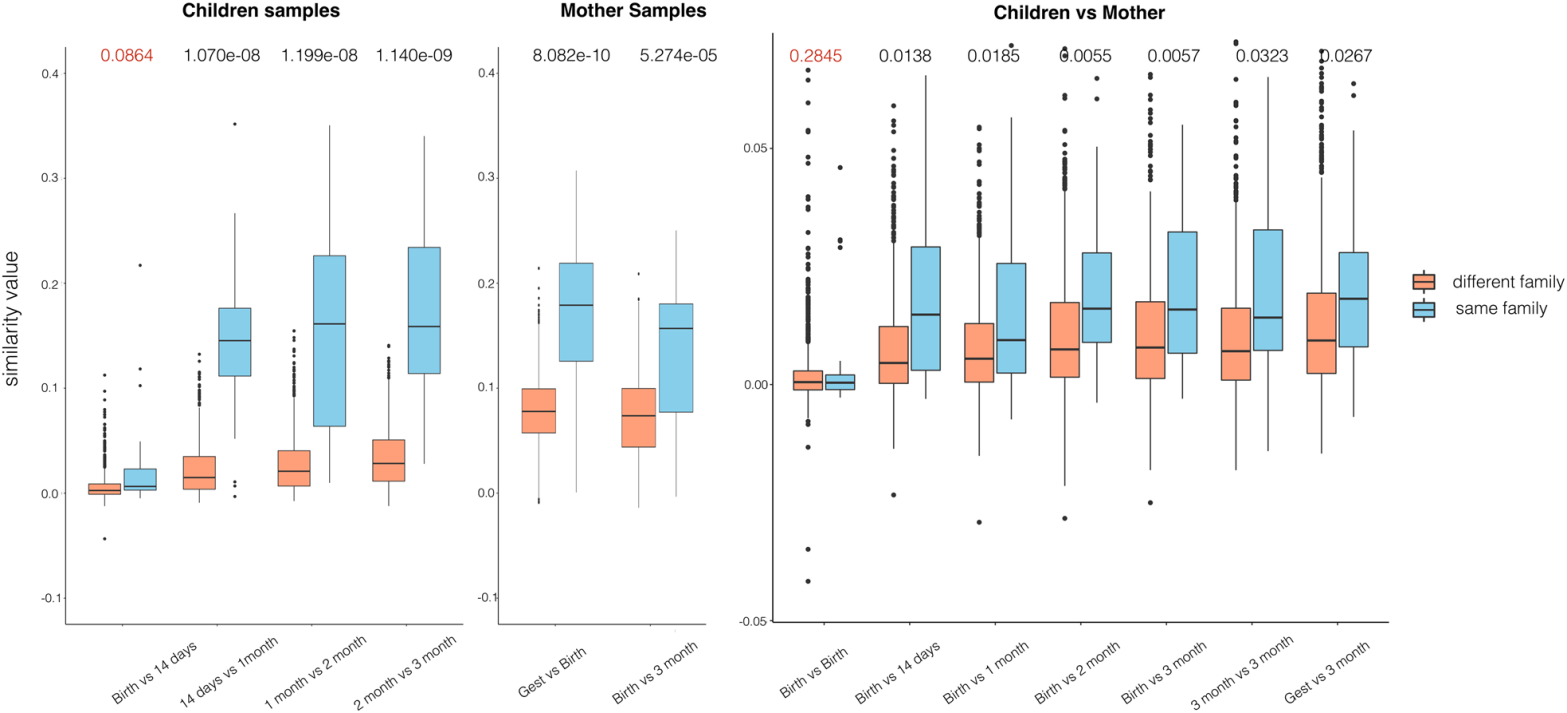
Similarity among temporal HGT networks at different time points for individual children (left), mothers (center), and child-mother paired samples (right). The network similarity is defined as $Jaccard\ similarity*degree\ correlation$, here $Jaccard\ similarity$ measures the similarity between node sets of two networks and $degree\ correlation$ measures the similarity on network topology

We measured the similarity of HGT networks to explore the evolvement of dynamic gut microbiota (see “Method” section). First, child HGT networks show individual similarity. We compared HGT networks between adjacent time points chosen from the same individual and random two individuals. The network similarity is measured by $$Jaccard\ similarity*degree\ correlation$$. As illustrated in Fig. 7, for child gut microbiota, HGT networks from the same child have significantly higher similarity over time than that from different children except for the first 2 weeks (Student’s t-test, $$p=0.0864, p=1.07e^{-8}, p=1.199e^{-8}, p=1.14e^{-9})).$$ This demonstrates the child form individual-specific gut microbiota after one month since birth. Second, for maternal gut microbiota, the HGT network maintains significant similarity between adjacent time points in the same individual compared to random paired HGT networks ($$p=8.082e^{-10}, p=5.274e^{-5}$$).

Third, to explore the transmission of the HGT network from mother to child, we compared maternal and child HGT networks within- and across-families. As illustrated in Fig. 7, we compared maternal HGT networks at birth to child HGT networks at birth, 14 days, 1 month, 2 month ($p=0.2845, p=0.0138, p=0.0185, p=0.0055$). HGT networks of a mother and her child have significant similarities than those from random two families except for maternal and child HGT networks at birth ($p=0.2845$). Besides, we also compared the child HGT network at three months to maternal HGT networks at gestational week 27, birth, and three months post-delivery ($p=0.0267, p=0.0057, p=0.0323$), These results show that there is significant similarity between HGT networks of a mother and her child. Therefore, the mother does pass along microbes harboring family-specific HGT events to her child, which leads to the similarity of gut microbiota between a mother and her child. This is a family-specific gut microbial similarity captured by their HGT networks.

**Fig. 8:**
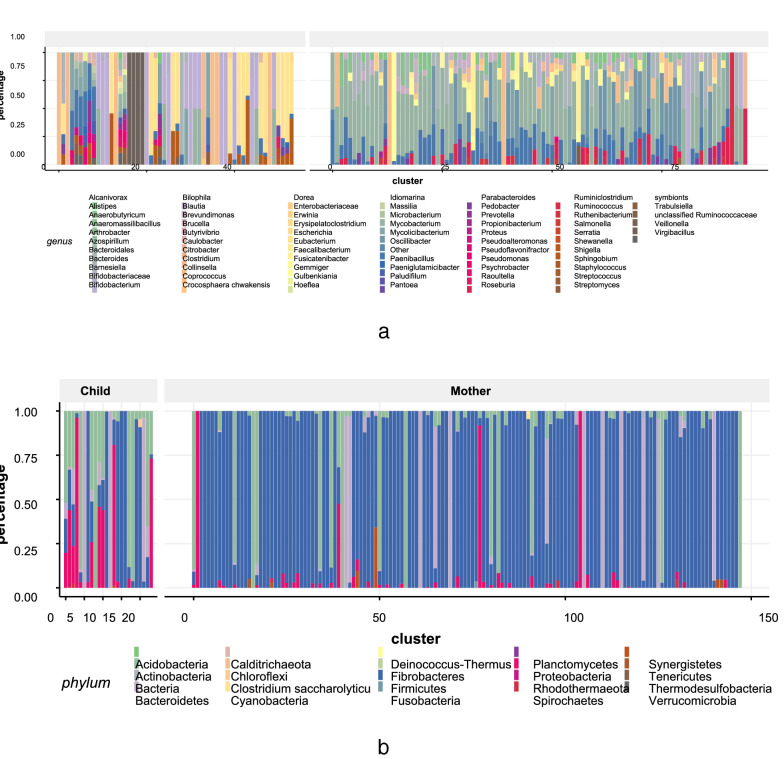
**a** Comparison between the composition of 54 child HECs and 95 maternal HECs at genus level; **b** Phylum composition of 24 child and 148 maternal clusters of HGT communities

#### Conserved edges in Mother-to-Child HGT network

We first focus on edges in the HGT network and identify multiple conserved edges corresponding to host states. In HGT network, the edge is constructed according to HGT events (see “Method” section). The existence of conserved edges across multiple samples implies that HGT events occurred in multiple samples. We analyze conserved edges by clustering HGT events. By applying cluster analysis to HGT events (see “Method” section), we found HGT events that occurred in similar samples form HGT event clusters (HECs). For each HEC, we determine its label according to the host state of samples to which the majority of HGT events belong (see “Method” section). Further analysis on HECs helps explain how different host states affect the genus composition of conserved edges.

We get 54 child HECs and 95 maternal HECs. Child and maternal HECs have different composition at genus level, see Fig. 8a. The genus composition of HECs shows that the *Bifidobacterium* is the genus that significantly different in child and mother (21.59% in Child vs. 1.82% in Mother, p-value = 2.1604$e^{-6}$), which is consistent with the finding in [48] that *Bifidobacterium* is the predominant bacteria in the child’s gut. Besides, we also found that *Escherichia* (20.24% in Child vs 0% in Mother, p-value = 1.6749$e^{-8}$), *Microbacterium* (6.48% in Child vs 33.7% in Mother, p-value = 1.1742$e^{-10}$), *Mycolicibacterium* (0% in Child vs 11.78% in Mother, p-value = 1.5939$e^{-4}$) are different in HECs of child and mother. *Escherichia* includes a number of pathogenic species such as *Escherichia coli* [49]. The high percentage of *Escherichia* contained in child-specific HECs is one common health risk to infants [50]. We have observed 13 other species of Escherchia in HECs of child including Escherichia sp. KTE172, Escherichia sp. 1_1_43, Escherichia sp. 4_1_40B, Escherichia sp. B1147, Escherichia sp. TW14182, Escherichia albertii, Escherichia sp. TW15838, Escherichia sp. KTE159, Escherichia fergusonii ATCC 35469, Escherichia sp. KTE52, Escherichia marmotae, Escherichia sp. TW10509, and Escherichia sp. TW09231.

#### HGT communities differ at different age state

To find out preserved local network structure in multiple HGT networks, we detected communities in each HGT network. These communities are defined as HGT communities. Then we identified and determined the label of HGT community clusters (HCCs) (see “Method” section).

We got 24 child and 148 maternal HCCs in the Mother-to-Child data set, and get the phylum composition of these HCCs as shown in Fig. 8b. The average phylum composition of child HCC is *Firmicutes*: 35.3%, *Actinobacteria*: 29.8%, *Proteobacteria*: 19.4%, *Bacteroidetes*: 15.1%, Others: 0.4%. The average phylum composition of maternal HCC is *Firmicutes*: 78.2%, *Actinobacteria*: 10.1%, *Bacteroidetes*: 7.9%, *Proteobacteria*: 3.2%, Others: 0.6%. Compared with child HCCs, the increasing of *Firmicutes* (p-value = 8.0091$e^{-7}$), the decreasing of *Proteobacteria* (p-value = 2.8785$e^{-5}$) and *Actinobacteria* (p-value = 0.0015) are significant. Genomes belonging to *Firmicutes* which plays an important role in maintaining the gut health [39] are the main maternal HCCs members. Communities in child HCCs are more diverse at the phylum level since child HGT network contains more nodes belonging to genus from *Proteobacteria* and *Actinobacteria*. As shown in the genus composition of child HECs, these genus include *Bifidobacterium*, *Escherichia*, etc.

### Analysis of longitudinal IBD HGT networks

#### Disease-specific similarity of individual HGT networks

**Fig. 9:**
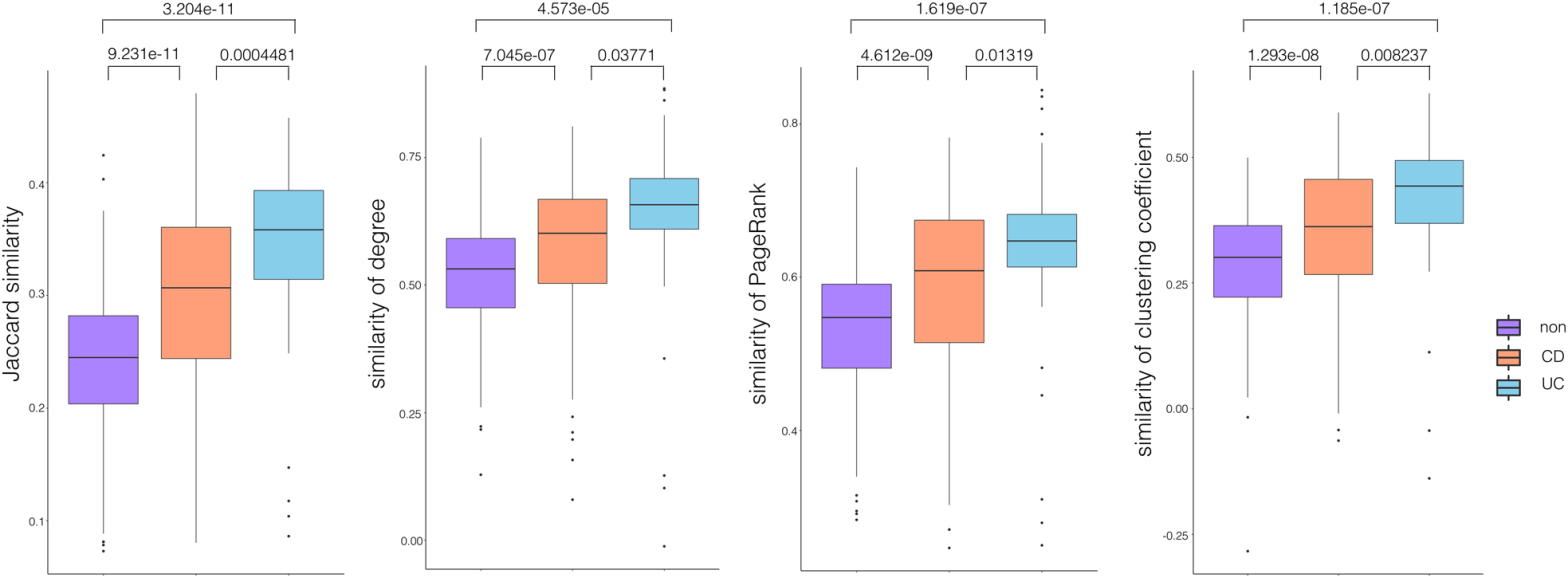
Compares the similarity among individual HGT networks at different time points in CD, UC, and non-IBD controls. The four similarity metrics are Jaccard index, degree correlation, pagerank correlation, and clustering coefficient

We also explored the HGT network similarity in different disease states using the longitudinal IBD data set. We utilize four similarity metrics, including Jaccard index, degree correlation, Pagerank correlation, and clustering coefficient, to measure network similarity. For each individual, we calculate the four metrics among his HGT networks at different time points (tables and heatmaps are in Additional files 1 to 5). Finally we get disease-specific similarity metrics in CD, UC, and non-IBD controls as shown in Fig. 9. The four similarity metrics in Non-IBD samples are significantly lower than those in IBD samples. The lower similarity among Non-IBD HGT networks at different time points indicates that healthy gut microbiota is more flexible than IBD gut microbiota.

#### Conserved edges in longitudinal IBD HGT network

**Fig. 10:**
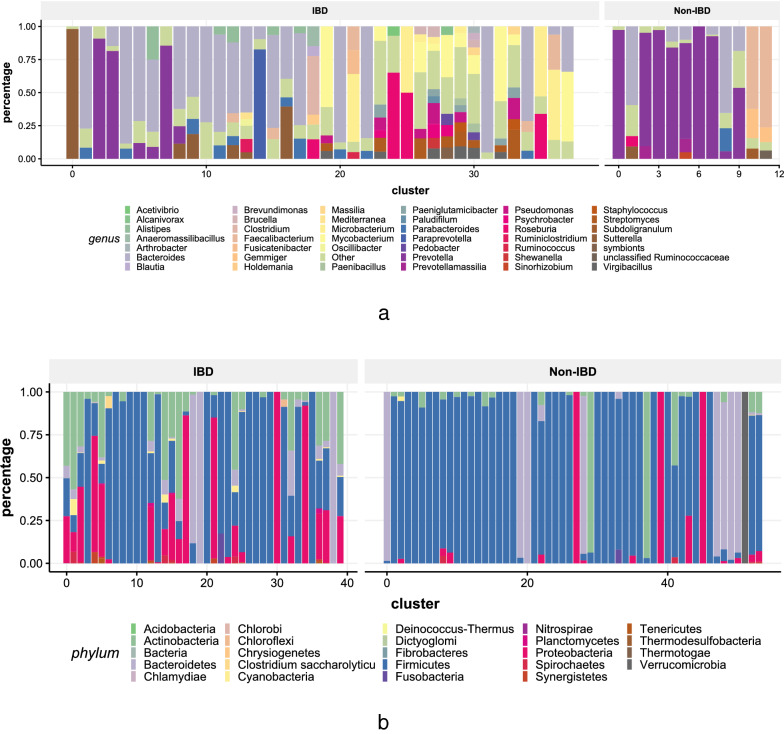
**a** Comparison between the composition of 38 IBD and 12 Non-IBD HECs at genus level; **b** Phylum composition of 40 IBD and 54 Non-IBD clusters of HGT communities

We get 38 IBD HECs and 12 Non-IBD HECs. Figure 10a compares the composition of these HECs at genus level. Compared with IBD HECs, more HGT events in Non-IBD HECs occur in Prevotella (57.4% in Non-IBD vs. 7.7% in IBD, p-value = 0.4527), which is a critical bacterium for healthy microbiota [51]. In contrast, more HGT events in dectected IBD HECs are contained in pathogenic genus such as *Mycobacterium* (0% in Non-IBD vs 7.18% in IBD, p-value = 0.2937), *Sutterella* (0.78% in Non-IBD vs 4.82% in IBD, p-value = 0.3894) [52], *Pseudomonas* (0% in Non-IBD vs 1.63% in IBD, p-value = 0.0006). These genera could be treated as potential biomarkers. *Pseudomonas* plays an important role in IBD [53]. These findings demonstrate that groups harbor group-specific edges (Additional files 2, 3, 4, 5). Host states affect the conservation of HGT edges. Identifying these conserved edges helps to locate the key HGT events under specific conditions.

#### HGT communities differ between the IBD and non-IBD individuals

We found 40 IBD and 54 Non-IBD HCCs in the longitudinal IBD datasets. Figure 10b shows the distribution of phylum composition of them. The figure implies that IBD HGT communities have different phylum composition with Non-IBD HGT communities. The average phylum composition of Non-IBD HCC is *Firmicutes*: 70.7%, *Bacteroidetes*: 14.4%, *Proteobacteria*: 6.8%, *Actinobacteria*: 5.9%, Verrucomicrobia: 1.9%, Others: 3%. The average phylum composition of IBD HCC is *Firmicutes*: 53.6%, *Proteobacteria*: 19.6%, *Actinobacteria*: 14.5%, *Bacteroidetes*: 9.9%, Others: 2.4%. Compared with Non-IBD HGT communities, IBD HCCs have less genome nodes from *Firmicutes* and *Bacteroidetes* which are the two most dominant phyla in the large intestine of healthy adults [39]. While the phylum composition of IBD-specific HGT communities have significant increasing of *Proteobacteria* (p-value = 0.0194) and *Actinobacteria* (p-value = 0.0316). Many species belonging to *Proteobacteria* and *Actinobacteria* have strong association with the pathogenesis of IBD [40, 41].

**Fig. 11:**
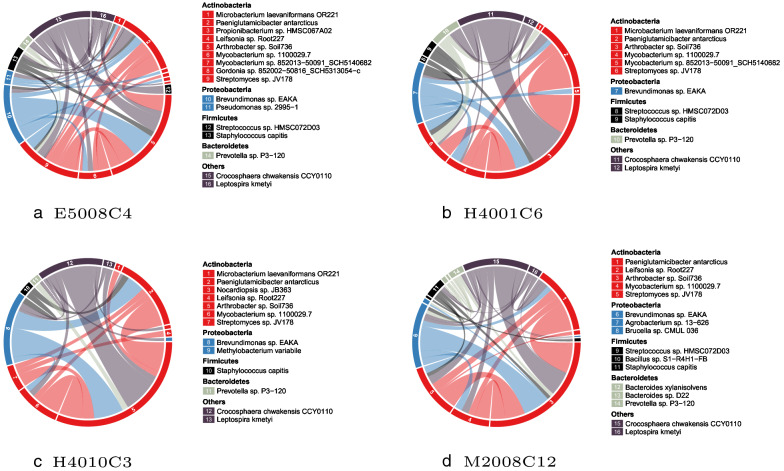
Four HGT communities from IBD community cluster 1. Segments in the circle represent microbial species. Linkage between two segments represents the number of detected HGT events. The four communities have similar structure and are all detected from IBD patients

Figure 11 illustrates the four similar communities from one IBD HCC. The four communities are detected from four different IBD patients: E5008C4, H4001C6, H4010C3, and M2008C12. They have a high percentage of overlapping species and share similar linking patterns between species. Since HGT communities in this cluster are mainly from the IBD group, their common structure can be used as a biomarker of IBD.

**Fig. 12:**
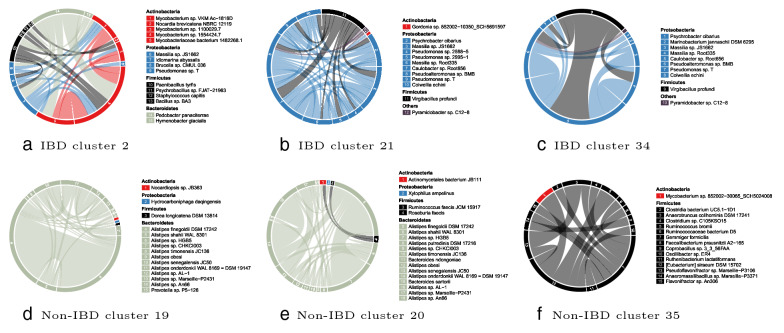
Six randomly selected communities from six different HGT community clusters. The three communities in the first row are from IBD clusters. The remaining three communities in the second row are from Non-IBD clusters. The two groups of communities are less similar compared to communities within the same group

As shown in Fig. 12, HCCs detected from IBD and Non-IBD possess communities with different composition. Figure 12a–c are three randomly selected communities from three IBD HCCs. Figure 12d–f are three randomly selected communities from three non-IBD HCCs. Compared to HGT communities from clusters with the same label, HGT communities from clusters with different labels share less common members and therefore are much more dissimilar.

Moreover, the species in the community of IBD HCC are found to be associated with IBD. Figure 12a contains species (*Mycobacterium* sp. VKM Ac-1816D, *Mycobacterium* sp. 1100029.7, and *Mycobacterium* sp. 1554424.7) from genus *Mycobacterium*, which includes pathogens known to cause IBD [41, 54, 55]. Figure 12b, c contains species (*Pseudomonas* sp. 2588-5, *Pseudomonas* sp. 2995-1, and *Pseudomonas* sp. T) belong to genus *Pseudomonas*, which is also associated with IBD [40].

#### Gene fusions associated with HGT events in Longitudinal IBD data set

HGT event can cause the combination of parts of two genes belonging to two different genome sequences to fusion a gene (see “Method” section). From the Longitudinal IBD dataset, we found multiple HGT-caused gene fusion events that are related to the HGT mechanism, as listed following. We detected 2186 gene fusion events, which involve 1280 genes and 800 different gene functions. Gene fusion events containing genes associated with the mechanism of HGT include *recombinase family protein* (51/2186) [56], *plasmid mobilization relaxosome protein Mob* (39/2186) [57], *IS110 family transposase* (23/2186) [58], *site-specific integras* (23/2186) [59], *conjugal transfer protein Tra* (20/2186) [60] and *transposase* (2/2186) [58]. Specifically, as shown in Table 1 in Appendix section, we detected 7 and 1 fusions of multidrug transporter genes in IBD and Non-IBD samples, respectively. The HGT event column denotes the two genome references for each HGT event. The number in the bracket denotes the HGT breakpoint position on the reference. Each HGT event consists of two HGT breakpoints. Fusion Gene A and B columns describe the information of the two fusion genes. The label column denotes the label of each sample. The multidrug transporter gene could encode multidrug transporters, which play an important role in multidrug resistance. By identifying and ejecting xenobiotic substances, multidrug transporters protect bacteria against antibacterial agents [61]. Products of the detected multidrug transporter genes involved in HGT events include *multidrug SMR transporter* [62], *multidrug transporter AcrB* [63], *multidrug transporter MatE* [64], and *multidrug efflux RND transporter permease subunit* [65].

## Method

### Reference genomes construction

To construct HGT networks, we downloaded all bacterial genomes from the National Center for Biotechnology Information (NCBI). For each taxonomy, we choose the genome with the minimal scaffold number and highest completeness, whose contamination is less than 10% in Genome Taxonomy Database (GTDB) taxonomy evaluation results [66]. Finally, we obtain 109,419 bacterial genomes, which consist of 16,093 species with 1,246,881 scaffolds.

### HGT network construction

**Fig. 13:**
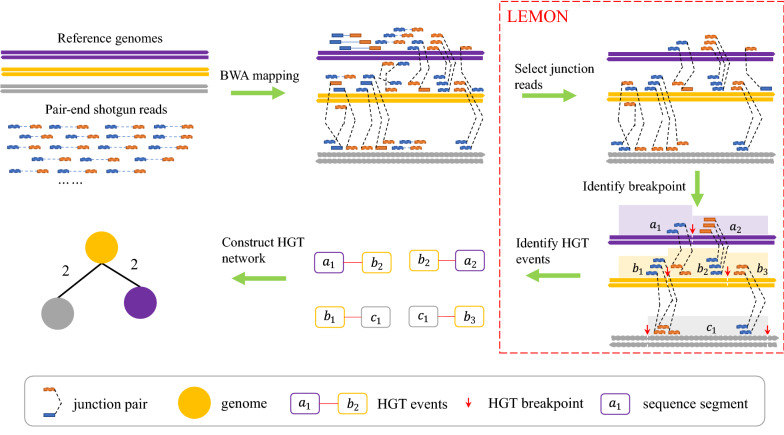
Overview of HGT network construction

Figure 13 illustrates the construction procedure of the HGT network from raw NGS data. Firstly, we utilized Burrows–Wheeler Aligner (BWA) to map paired-end reads against reference genomes. Then we took the aligned reads as the input of LEMON [15] to detect HGT breakpoints on reference genomes. According to the HGT breakpoints, the genome sequences are split to segments, such as $a_1$, $a_2$, $b_1$, and so on. One HGT event is defined as the linkage of two segments belonging to two different genome sequences due to HGT, such as $(a_1,b_2)$, $(a_2,b_2)$, $(b_2,c_2)$ in Fig. 13. In order to construct an HGT network, for any two reference genomes, if there exist HGT events between them, the two reference genomes are treated as nodes and linked by an HGT event. The weight of an edge is the number of HGT events between the two reference genomes. The three steps in the red box are key parts of LEMON, which are described in detail in [15].

### Evaluate the goodness of fit for power-law distribution

The scale-free network has its degree distribution $P_k$ follows a power law $P_k \sim k^{-\alpha }$, where *k* is degree and $\alpha$ is degree exponent whose value typically satisfies $2<\alpha <3$. As suggested in [43], in order to evaluate the goodness of fit for the HGT network to power-law distribution, we compared it with the other three heavy-tail distributions: exponential distribution, Weibull distribution, and lognormal distribution with positive parameter $\mu$. Let $D={d_1,\ldots ,d_n}$ denote the node degree set of HGT network, $\forall d_v \in D$, we have $d_v\ge 1$ since an HGT event links two reference genomes, which means a genome node in HGT network should link at least another node. So the median of D has $median(D)\ge 1$. As for distribution $Lognormal(\mu , \sigma ^2)$, its median is $exp(\mu)$. Therefore, if we use $Lognormal(\mu , \sigma ^2)$ to fit *D*, we must have $exp(\mu)\ge 1$, which means $\mu \ge 0$. We use the log-likelihood ratio test [42] to compare the goodness of two fits. In our experiments, for each HGT network, we performed three tests: power-law vs. exponential, power-law vs. lognormal_positive, and power-law vs. Weibull. In each test, if the ratio is larger than 0, the power-law achieves a better fit than the other.

### Von Newman entropy

Structural complexity is an important characteristic of complex networks. It greatly determines the function and status of complex networks. The growth of temporal networks often leads to structural change, which also means a change of complexity. Ye et al. [67] proposed an approximation of von Neumann entropy to measure the complexity of dynamic networks. Since the simplified von Neumann entropy can be interpreted as the thermodynamic entropy of the network, we can describe the complex dynamic system from the perspective of statistical thermodynamics. Therefore, we applied the simplified von Neumann entropy to measure the complexity of temporal HGT networks. Let *H*(*V*, *E*) denote the HGT network, *V* denotes the vertex set, which represents genomes linked by HGT events, *E* is an edge set, which indicates whether there exist HGT events between two genome nodes. Then the von Neumann entropy $H_{VN}$ is defined as follows,

1 $$\begin{aligned} H_{VN}=1-\frac{1}{|V|}-\frac{1}{|V|^{2}}\sum _{(u,v)\in E} \frac{1}{d_ud_v} \end{aligned}$$

Here $d_u$ is the degree of node *u*. |V| is the number of nodes, (u,v) is an edge in *E*.

### HGT event rate

We take next-generation sequencing (NGS) short reads as input of LEMON to detect HGT events on reference genomes. The amount of raw data and the length of reference genomes can affect the number of detected HGT events in one sample We normalized the number of HGT events and define the normalized number as *HGT event rate*. Let *H* denotes the number of detected HGT events in sample *S*, $G=\{g_1,g_2,\ldots ,g_N\}$ is the set of genomes linked by HGT events. Denote $r_i$ as the number of reads uniquely mapped onto genome $g_i$, $g_i\in G$, and denote $l_i$ is the length of $g_i$. So we get two sets $R=\{r_1,r_2,\ldots ,r_N\}$ and $L=\{l_1,l_2,\ldots ,l_N\}$. The HGT event rate ${\bar{H}}$ is calculated as follows

2 $$\begin{aligned} {\bar{H}}=\ln {\frac{H}{\sum _{i=1}^Nr_i/\sum _{i=1}^Nl_i}} \end{aligned}$$

here $\sum _{i=1}^Nr_i/\sum _{i=1}^Nl_i$ is approximate to the average read depth. ${\bar{H}}$ represents the logarithm of the rate between the number of HGT events and the read depth.

### Similarity metric for HGT networks

After obtaining the HGT networks, we calculated the similarity between networks according to the following measurements. First, we measured the similarity between networks by Jaccard similarity of species that present in the network. If two HGT networks share more species, then they obtain higher Jaccard similarity. Besides species present and absent, we also measured network similarity based on the topology property for species in networks, e.g., degree, PageRank, clustering coefficient. For two networks, we find species shared by both networks, record their degree in two networks, and calculate the Spearman correlation on the two-degree lists. The generated correlation implies the consistency of the importance rank for every species in two networks at the aspect of different topology properties. Finally, we assigned these comparison pair into multiple groups and studied the similarity distribution. To test whether the similarity between networks in the same family is significantly higher than it between different families, we applied a T-test to the similarity values between these two groups in different sample times.

### Detecting and clustering HGT communities

We detected community in each HGT network in each sample and to study the community evolution by comparing those detected HGT communities across time/individual. First, for community detection, we applied the Leiden algorithm [68], which is based on the Louvain algorithm. Louvain algorithm is a popular community detection methods which optimize the modularity in the network by local move and aggregate network iteratively. However, it can generate badly connect communities sometimes. Leiden overcomes it by adding a smart local move to refine the partition of nodes in each iteration. Therefore Leiden generates more robust and well-connected communities, which is a better solution in our situation. After community detection, we further clustered HGT communities detected in all different individuals and different times. By applying clustering analysis on communities, we can find common communities across samples and calculate their composition within a group. We call the common community group as HGT community clusters (HCCs). For clustering, the distance between communities is calculated from their Jaccard similarity; then, we apply a hierarchical cluster on all identified communities to find HCCs.

### HGT event cluster

For each HGT event, we recorded the number of samples it present. HGT events that show in less than the minimum sample threshold are filtered out. The minimum samples threshold is set as 5 and 4 in Mother-to-Child and Longitudinal IBD experiments, respectively. Then we measured the relationship between HGT events by their Jaccard similarity considering the samples they show up. After convert similarity to distance, we applied hierarchical clusters and determined classes by dynamic tree cut. We set distance to 0.6 and minimum class sizes as 10 and 20 for Mother-to-Child and Longitudinal IBD data sets, respectively. The outcome of the hierarchical result is HGT event clusters (HECs). Each HEC refers to the group of HGT events which occur together.

### Label clusters of HGT communities and events

Since each cluster of HGT communities/events (HCC/HEC) consists of HGT communities/events from different groups, it may contain multiple labels with an unequal number of communities/events. The label of the cluster should be determined by the predominant label in the cluster. Therefore we determined the label of cluster as following. Without loss of generality, we take the determination of labels for HCCs in longitudinal IBD data set as an example. From longitudinal IBD data set, we got 94 HCCs $$\{cluster_i=[c_{i1},\ldots ,c_{in}], i=1,\ldots ,94\}$$, here $$cluster_i$$ represents the *i*th HCC, $$c_{i\cdot }$$ denotes the communities in $$cluster_i$$. According to the group of the sample from which the community is detected, each community is labeled as Non-IBD or IBD. For community $$c_{i\cdot }$$, we denote $$l(c_{i\cdot })$$ as its label. We counted the number of communities belonging to different labels and let $$COUNT_i(Non-IBD)$$ and $$COUNT_i(IBD)$$ represent the number of communities having label $$Non-IBD$$ and *IBD* respectively in $$cluster_i$$. If $$COUNT_i(Non$$-$$IBD)>COUNT_i(IBD)$$, then $$l(cluster_i)=Non$$-*IBD*. Otherwise $$l(cluster_i)=IBD$$. However, the number of Non-IBD samples *num*(*Non*-*IBD*) is 38 and the number of IBD samples *num*(*IBD*) is 109, the larger value of *num*(*IBD*) make $$cluster_i$$ tend to contain more communities with IBD labels. To correct the bias, we set $$l(cluster_i)$$ as follows,

3 $$\begin{aligned} l(cluster_i)= {\left\{ \begin{array}{ll} \text {Non-IBD} &{} {\frac{COUNT_i(Non-IBD)}{num(Non-IBD)}>\frac{COUNT_i(IBD)}{num(IBD)}}\\ \text {IBD} &{} {\frac{COUNT_i(Non-IBD)}{num(Non-IBD)}<\frac{COUNT_i(IBD)}{num(IBD)}} \end{array}\right. } \end{aligned}$$

Here $\frac{COUNT_i(Non-IBD)}{num(Non-IBD)}$ and $\frac{COUNT_i(IBD)}{num(IBD)}$ represent the relative amount of $Non-IBD$ and *IBD* communities that are contained in $cluster_i$ respectively. If $\frac{COUNT_i(Non-IBD)}{num(Non-IBD)}<\frac{COUNT_i(IBD)}{num(IBD)}$, it means that HGT communities sharing common structure in $cluster_i$ are mainly from IBD samples and their common structure could act as a candidate biomarker of IBD.

Let $[g^1_j,...,g^m_j]$ denotes the genomes set of community $c_j$. For each cluster of communities $cluster_i=[c_{i1},...,c_{in}]$, we collect its communities’ genomes together and get the genome set $G_i=[g^1_{i1},...,g^{m_{i1}}_{i1},...,g^{m_{ij}}_{ij},...,g^{m_{in}}_{in}]$ for $cluster_i$, here $m_{ij}$ is the number of genomes in community $c_{ij}$. The phylum/genus to which each genome in $G_i$ belongs could be found on NCBI. Then, for $cluster_i$, we get the phylum set $P_i=[phylum(g^1_{i1}),...,phylum(g^{m_{i1}}_{i1}),...,phylum(g^{m_{ij}}_{ij}),...,phylum(g^{m_{in}}_{in})]$. Finally, we figured out the composition of $cluster_i$ under different conditions at the phylum level by calculating the percentage of each phylum in $P_i$. For each cluster of HGT events, we collect its genus set and figure out the percentage of each genus.

### Gene fusions in HGT events

We further extended the HGT network study to gene function.

**Fig. 14:**
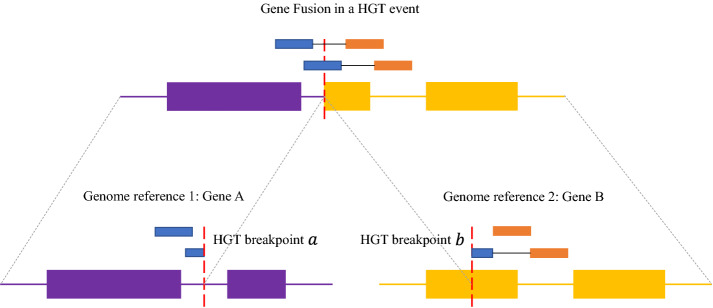
Gene fusions in one HGT event

Figure 14 denotes one fusion gene in an HGT event. The fusion gene contains two gene parts belonging to Gene A on genome reference 1 and Gene B on genome reference 2 respectively and *a* and *b* are the two HGT breakpoints. Since the two HGT breakpoints are in two gene regions, the combination of parts of two genes caused by HGT forms a fusion gene. We detected HGT breakpoints by LEMON and identified the gene fusion. So we can link the HGT event to gene function. The gene annotations (including Gene ID, start and end positions, function description) are collected from NCBI.

## Discussion and conclusion

HGT is the process of sharing genetic material among different microbial species. It links different species by transferring genetic information. By analyzing HGT networks constructed from two longitudinal metagenomic sequencing data sets: Mother-to-Child and longitudinal IBD data sets, we found the HGT network is scale-free, whose degree distribution follows a power law. Most nodes have a small degree and are connected by hub nodes. Moreover, statistics support the ultra-small world property of the HGT network. The distance between two randomly nodes in the HGT network could maintain small despite the growth of the network. Such a stable internal structure demonstrates the robustness of the HGT network in the everchanging environment. The HGT network also provides us an efficient way to model human gut microbiota. The development of the child gut microbiota during the first three months after birth could be captured by the evolvement of temporal HGT networks. The increasing of HGT network complexity and size is led by the growth of strains harboring HGT events. Furthermore, we have found a significant similarity between the family-specific child and maternal HGT networks. Therefore, HGT network could characterize bacterial transmission patterns from mother to child. It also demonstrates that maternal gut bacteria may be an essential source of child gut bacteria.

Analysis of the HGT community and HGT event demonstrates that age and disease change the internal structure of HGT networks. Compared with healthy individuals, the inflammatory conditions of the gastrointestinal tract in IBD patients are suitable for the growth of bacteria from *Proteobacteria* and *Actinobacteria* phylum. So HGT communities contain more bacteria from *Proteobacteria* and *Actinobacteria* and fewer bacteria from *Firmicutes*. Similar change is also observed in newborn children. By clustering HGT communities, we could find similar communities across multiple HGT networks that are from the same group. These similar communities reflect the influence of a specific host state on the structure of the HGT network and could be treated as potential biomarkers. The formation of the HGT community cluster is led by similar HGT events across samples. Through clustering HGT events, we realized that in IBD patients, the composition change of HGT communities is led by the increasing of HGT events mainly contained in the pathogenic genus *Mycobacterium*, *Sutterella*, and *Pseudomonas*. While in newborn children, we observed the increase of HGT events contained in *Bifidobacterium* and *Escherichia*. These differences reflect the alteration of gut microbiota in different conditions. The summarization of fusion genes in HGT events helps us better realize genes associated with HGTs. Many fusion genes in HGT events encode proteins, including recombinase family protein, plasmid mobilization relaxosome protein Mob, conjugal transfer protein Tra, and so on, that facilitate the horizontal transfer of genetic material. Furthermore, in IBD patients, more fusion genes caused by HGT events encode multidrug transporter proteins. This reveals that beneficial HGT events contribute to the survival of associated microbial strains in the gut under the specific selection pressure.

In summary, through the HGT network, we research human gut microbiota from a systematic perspective. The network analysis of Mother-to-Child and longitudinal IBD data sets demonstrate the characteristic of HGT networks differs under different conditions. In the future, we will apply our pipeline to analyze more HGT networks. It helps us get a deeper understanding of the relationship between host states and microbial interactions.

### Supplementary information

**Additional file 1.** Spearman correlation between degrees of nodes in HGT networks in IBD dataset.

**Additional file 2.** Jaccard similarity of nodes in HGT networks in IBD dataset.

**Additional file 3.** Spearman correlation between PageRank of all nodes in HGT networks in IBD dataset.

**Additional file 4.** Spearman correlation between the clustering coefficient of all nodes in HGT networks in IBD dataset.

**Additional file 5.** The heatmaps of similarity matrix for IBD HGT networks measured using Jaccard similarity (Fig. 1), Spearman correlation between degrees (Fig. 2), Spearman correlation between PageRank (Fig. 3), and Spearman correlation between the clustering coefficient (Fig. 4). The heatmap of similarity matrix for Infant HGT networks measured using Jaccard similarity*degree correlation (Fig. 5).

## Data Availability

283 Metagenomic samples were deposited to Sequence Read Archive (BioProject: PRJNA475246). 148 Metagenomic samples were deposited to Sequence Read Archive (BioProject:PRJNA389280). HGT detection package LEMON is available at https://github.com/lichen2018/LEMON. All analysis codes are freely available at https://github.com/lichen2018/HGT-network.

## Appendix

### Multidrug transporter gene fusions in HGT events

**Table 1:** Multidrug transporter gene fusions in HGT events

HGT event	Gene A		Gene B		Label
	Gene symbol	Description	Gene symbol	Description	
NZ_DS362246.1(124896) NZ_DS499674.1(613633)	BACUNI RS1296	Multidrug transporter	BACSTE RS1143	Hypothetical protein	CD
NZ_GG697149.2(303360) NZ_GG697156.2(32286)	FAEPRAA2165 RS0137	Multidrug SMR transporter	FAEPRAA2165 RS1330	Stage II sporulation protein	CD
NZ_DS499665.1(25120) NZ_DS499672.1(423580)	BACSTE RS0155	Hypothetical protein	BACSTE RS0626	MexE family multidrug efflux RND transporter periplasmic adaptor subunit	Non
NZ_DS499676.1(402917) NZ_DS499675.1(12487)	BACSTE RS1390	Hybrid sensor histidine kinase/response regulator	BACSTE RS1181	Multidrug transporter AcrB	CD
NZ_DS499672.1(447434) NZ_DS499662.1(199650)	BACSTE RS0636	ATP-dependent Clp protease Clp	BACSTE RS0090	Multidrug transporter MatE	UC
NZ_DS499671.1(180331) NZ_DS499673.1(122374)	BACSTE RS0406	Glycoside hydrolase family	BACSTE RS0707	Multidrug transporter AcrB	UC
NZ_DS499671.1(61003) NZ_DS499673.1(330329)	BACSTE RS0350	Hypothetical protein	BACSTE RS0784	Multidrug efflux RND transporter permease subunit	UC
NZ_DS499673.1(123680) NZ_DS499677.1(203460)	BACSTE RS0707	Multidrug transporter AcrB	BACSTE RS1579	UDP-3-O-[3-hydroxymyristoyl] N-acetylglucosamine deacetylas	UC
