# Supplementary material for: Understanding Horizontal Gene Transfer network in human gut microbiota
Source: Gut Pathog. 2020 Jul 9;12:33. doi: 10.1186/s13099-020-00370-9 (PMC7346641; doi:10.1186/s13099-020-00370-9)
Supplement: Supplementary file 5 — Additional file 5. The heatmaps of similarity matrix for IBD HGT networks measured using Jaccard similarity (Fig. 1), Spearman correlation between degrees (Fig. 2), Spearman correlation between PageRank (Fig. 3), and Spearman correlation between the clustering coefficient (Fig. 4). The heatmap of similarity matrix for Infant HGT networks measured using Jaccard similarity*degree correlation (Fig. 5). [file 13099_2020_370_MOESM5_ESM.pdf]

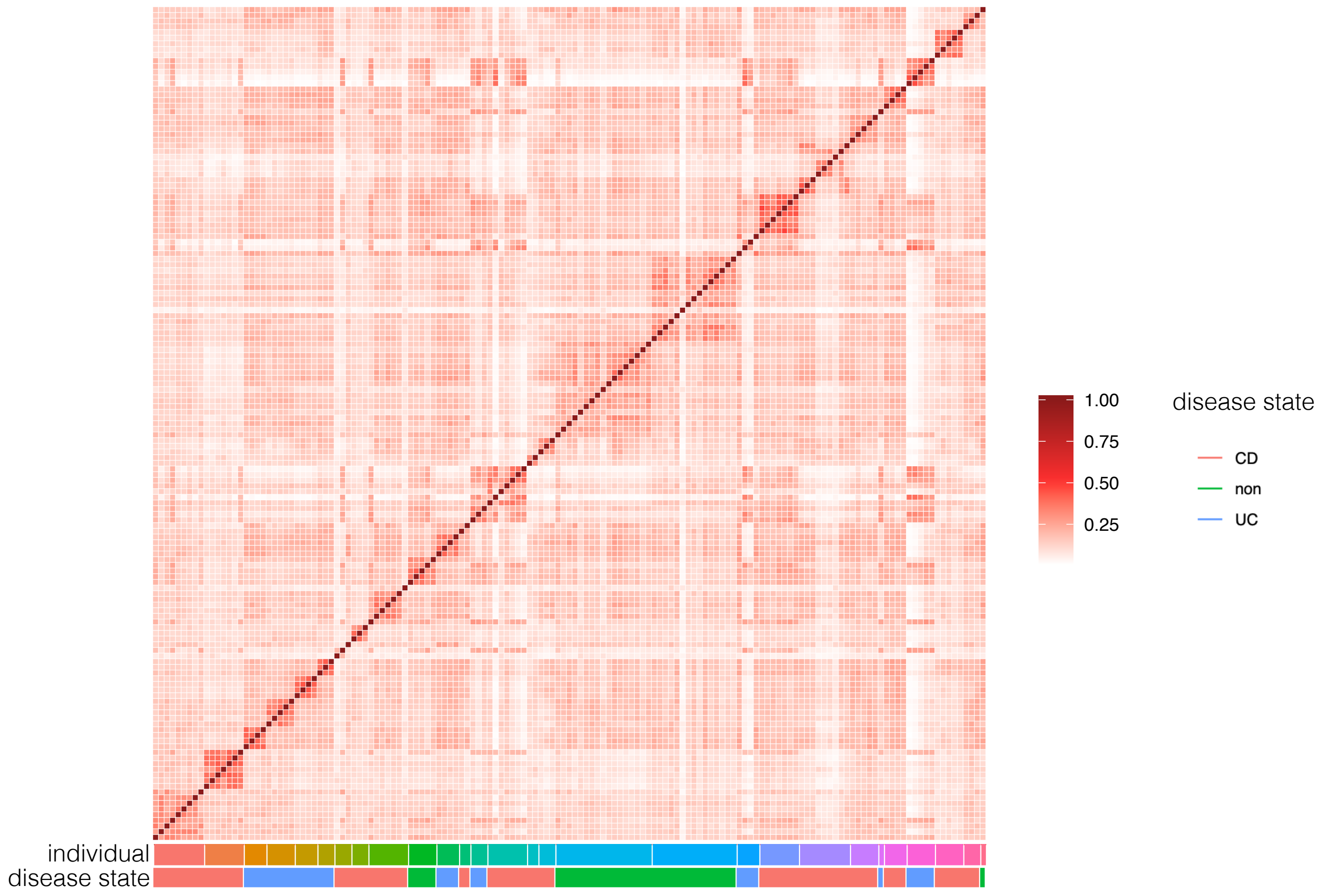

Additional file 5 Fig.1. Heatmap of IBD networks Jaccard similarity.

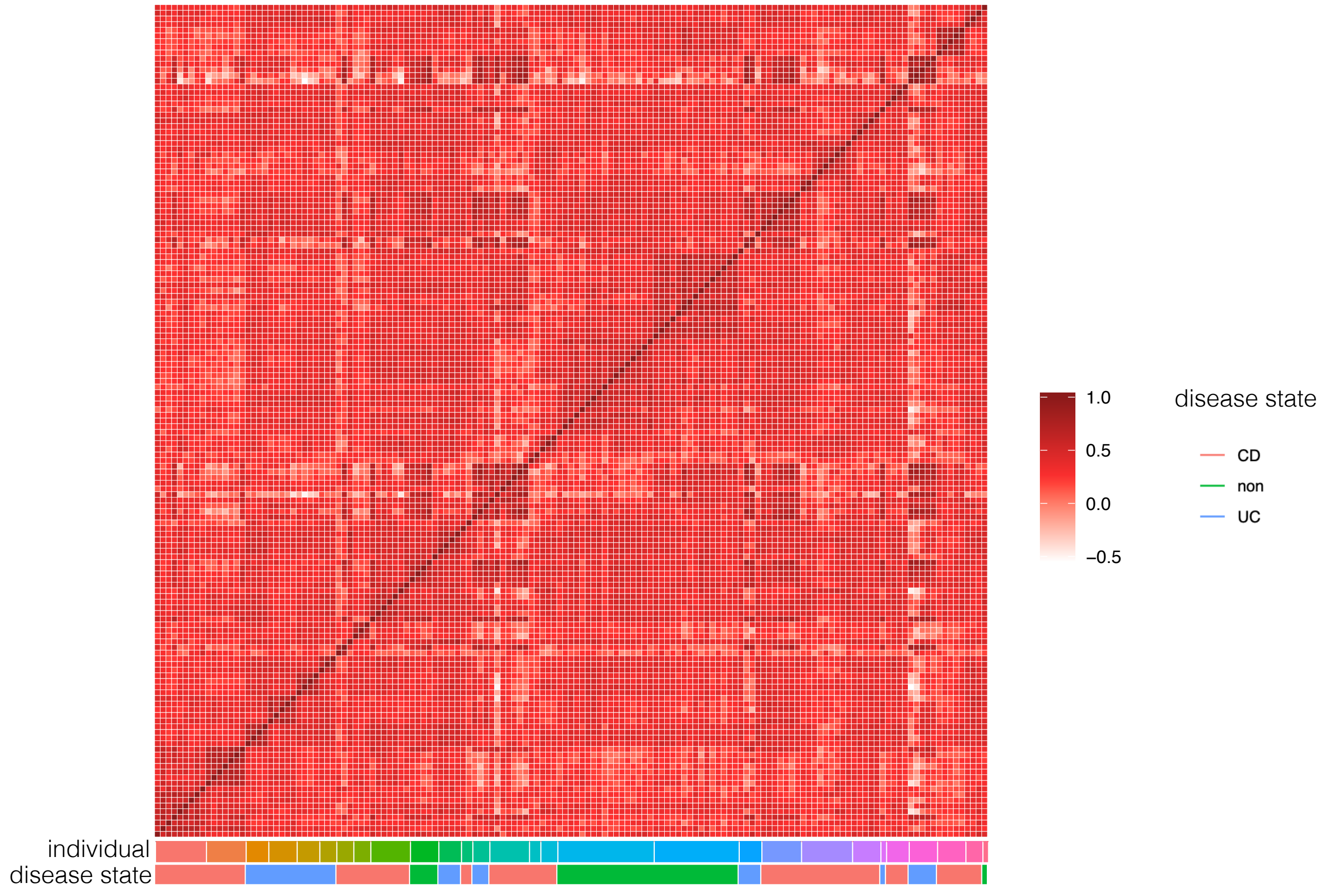

Additional file 5 Fig.2. Heatmap of IBD networks degree similarity.

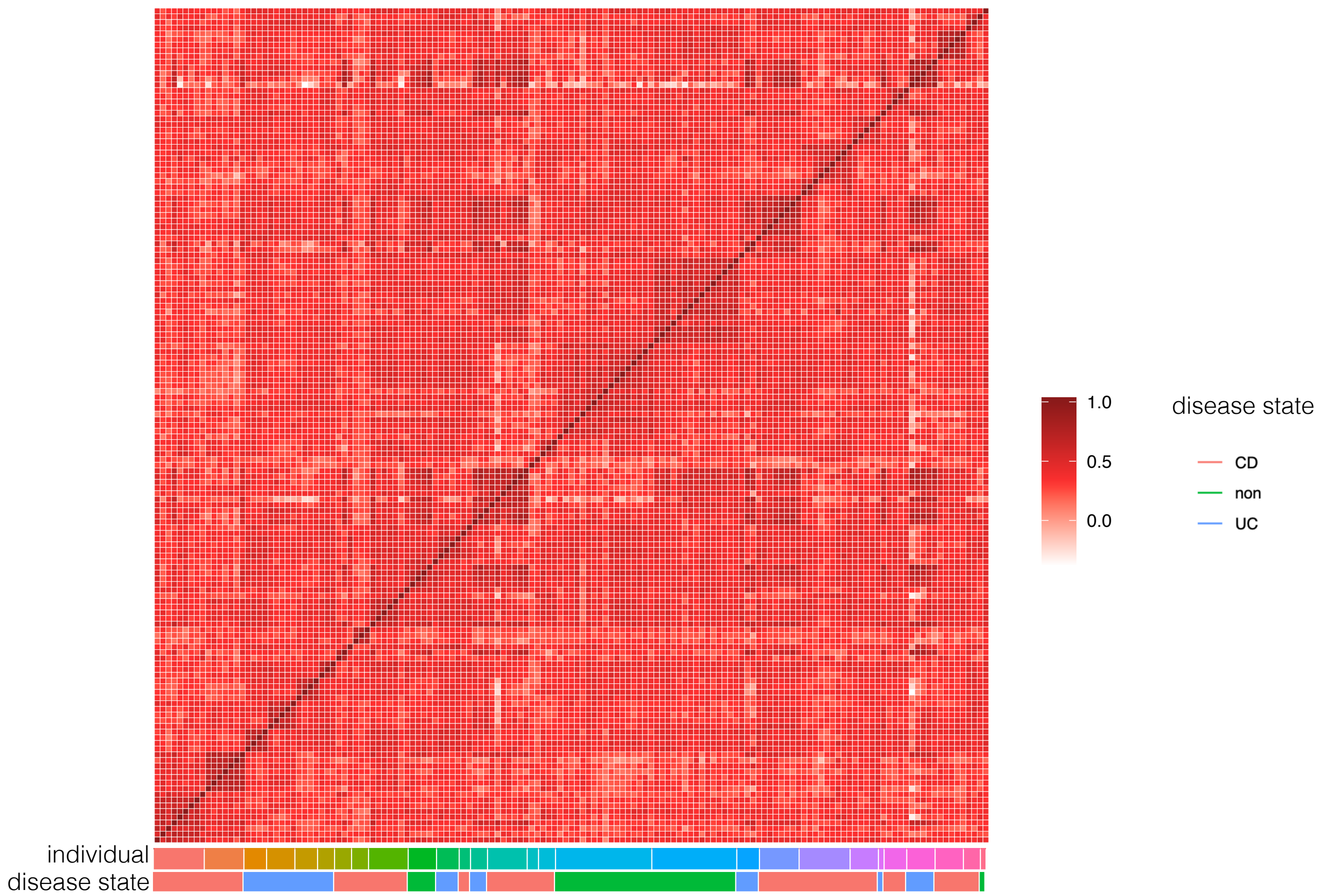

Additional file 5 Fig.3. Heatmap of IBD networks PageRank similarity.

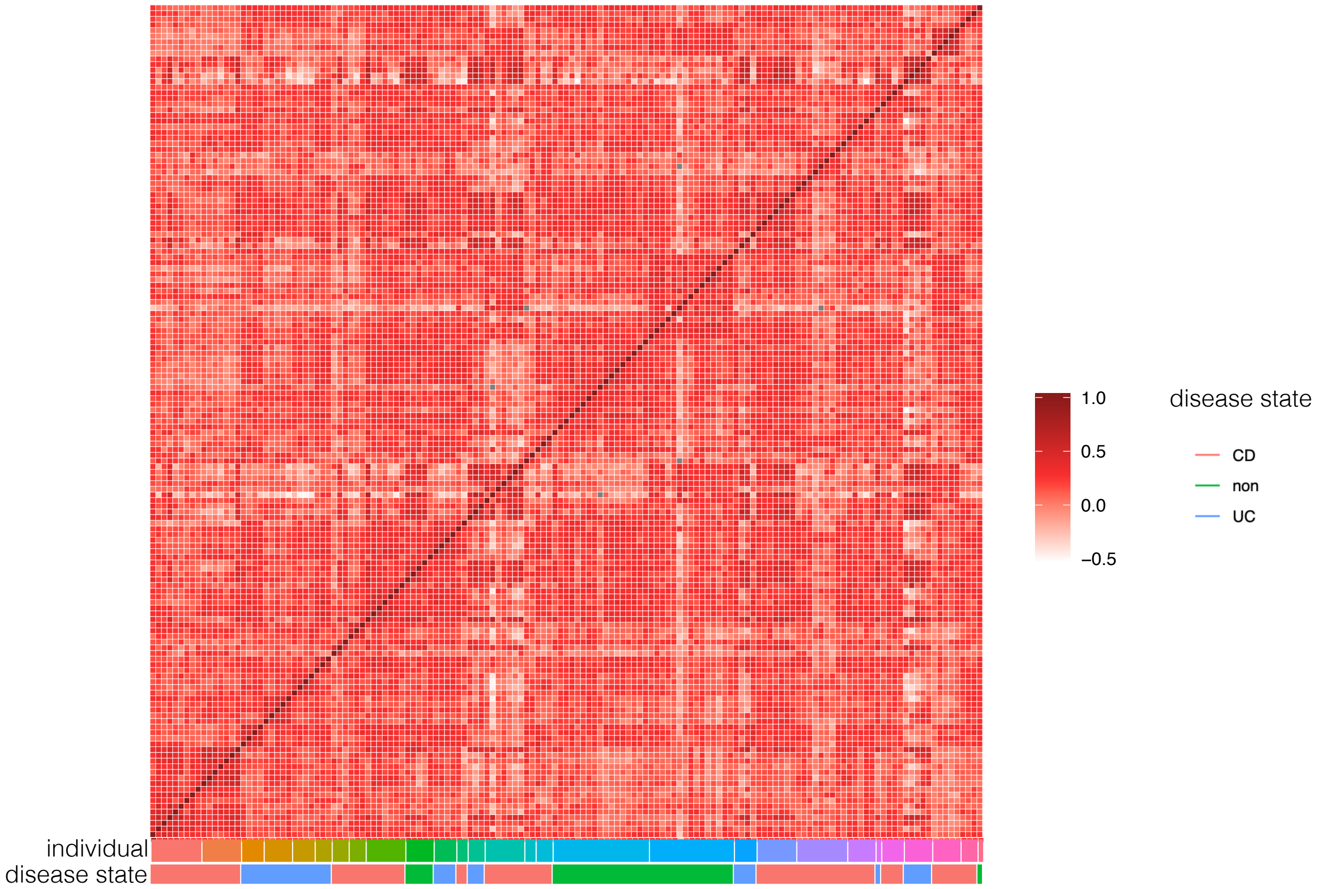

Additional file 5 Fig.4. Heatmap of IBD networks clustering coefficient similarity.

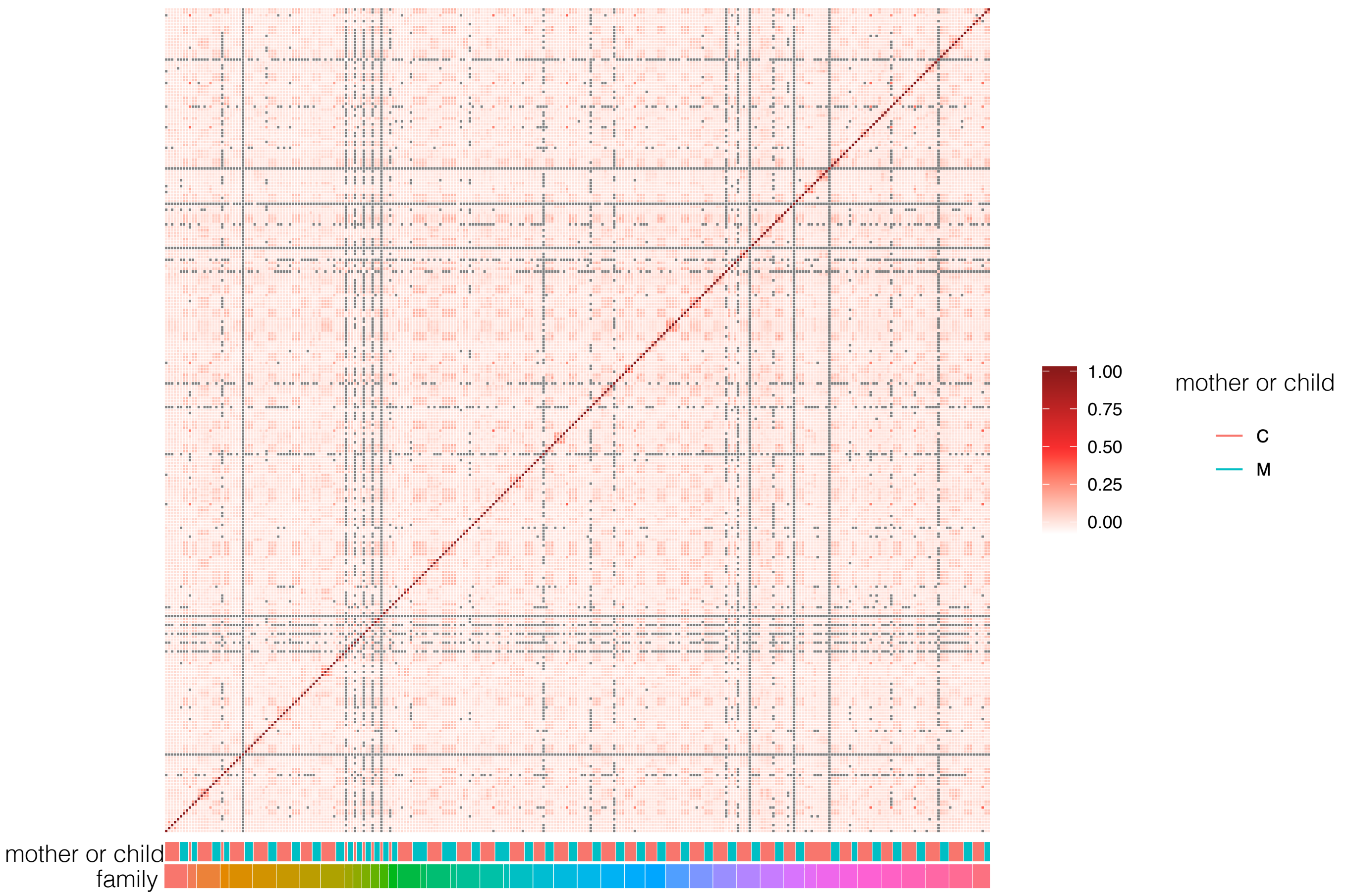

Additional file 5 Fig.5. Heatmap of Infant networks similarity. The network similarity is defined as Jaccard similarity*degree correlation
